# Quantitative Determination of 3-*O*-Acetyl-11-Keto-β-Boswellic Acid (AKBA) and Other Boswellic Acids in *Boswellia sacra* Flueck (syn. *B. carteri* Birdw) and *Boswellia serrata* Roxb

**DOI:** 10.3390/molecules21101329

**Published:** 2016-10-06

**Authors:** Giuseppe Mannino, Andrea Occhipinti, Massimo E. Maffei

**Affiliations:** 1 Biosfered S.r.l., Innovation Centre, Academic Spin-Off of the University of Turin, Via Quarello 15/A, Turin 10135, Italy; giuseppe.mannino@unito.it (G.M.) andrea.occhipinti@unito.it (A.O.); 2 Department of Life Sciences and Systems Biology, Innovation Centre, University of Turin, Via Quarello 15/A, Turin 10135, Italy

**Keywords:** Acetyl-11-Keto-β-Boswellic Acid, 11-Keto-beta-Boswellic Acid, boswellic acids, standardization, *Boswellia serrata*, *Boswellia sacra*, HPLC-DAD-ESI-MS/MS

## Abstract

*Boswellia serrata* and *Boswellia sacra* (syn. *B. carteri*) are important medicinal plants widely used for their content of bioactive lipophilic triterpenes. The qualitative and quantitative determination of boswellic acids (BAs) is important for their use in dietary supplements aimed to provide a support for osteoarthritic and inflammatory diseases. We used High Performance Liquid Chromatography (HPLC)-Diode Array Detector (DAD) coupled to ElectroSpray Ionization and tandem Mass Spectrometry (ESI-MS/MS) for the qualitative and quantitative determination of BAs extracted from the gum resins of *B. sacra* and *B. serrata*. Limit of detection (LOD), limit of quantification (LOQ), and Matrix Effect were assessed in order to validate quantitative data. Here we show that the BAs quantitative determination was 491.20 g·kg^−1^ d. wt (49%) in *B. sacra* and 295.25 g·kg^−1^ d. wt (30%) in *B. serrata*. Lower percentages of BAs content were obtained when BAs were expressed on the gum resin weight (29% and 16% for *B. sacra* and *B. serrata*, respectively). The content of Acetyl-11-Keto-β-Boswellic Acid (AKBA) was higher in *B. sacra* (70.81 g·kg^−1^ d. wt; 7%) than in *B. serrata* (7.35 g·kg^−1^ d. wt; 0.7%). Our results show that any claim of BAs content in either *B. sacra* or *B. serrata* gum resins equal to or higher than 70% or AKBA contents of 30% are simply unrealistic or based on a wrong quantitative determination.

## 1. Introduction

The genus *Boswellia* (Burseraceae), comprises 25 species of trees and shrubs which are widely spread in Arabia, the north-eastern coast of Africa and India [1]. Since ancient times, the natural resin of Boswellia trees has been collected and used to produce the oleo gum resin, frankincense (olibanum). The gum resin is harvested from incisions made on the trunk of the tree and the darkening of resin droplets is an index of oxidation [2]. Among *Boswellia* species, only a few are of economic importance as a natural source of phytopharmaceutical compounds, including *B. serrata* Roxb. and *B. sacra* Flueck (syn. *B. carteri* Birdw, syn. *B. undulatocrenata* Engl.) [3,4,5].

*B. serrata* is used for the treatment of oxidative and inflammatory damage [2], rhinitis [6] asthma [7], age-related disorders [8], neurorecovery [9], arthritis [10], skin disorder [11], cancer [12], and against several human pathogenic and plant pathogenic fungi [13]. Recently, the pharmacological properties and clinical effectiveness of *B. serrata* have been studied systematically [3].

*B. sacra* oleo gum resin is used in the treatment of gastric and hepatic disorders [14], skin disorders [15], for its hepatoprotective activity [16], analgesic effect [4], antiglycation and antioxidant activities [17], tumor suppression [18], anticoagulation effects [19], antinflammatory activity [20], and cardioprotective effects [21].

The main constituents of *B. serrata* and *B. sacra* are volatile oils, composed of monoterpenes and sesquiterpenes [22,23], diterpenes including incensole, incensole acetate and cembrenol (serratol) [24], lipophilic pentacyclic triterpene acids of the oleanane-(α-boswellic acids), ursane-(β-boswellic acids) and lupane-type (lupeolic acids), as well as an ether-insoluble fraction containing polysaccharides (arabinose, galactose, xylose) [25]. Among triterpenoids, bioactive boswellic acids are of particular interest, particularly 3-*O*-Acetyl-11-Keto-β-Boswellic Acid (AKBA), 11-Keto-beta-Boswellic Acid (KBA), and the various β-boswellic acids (βBAs), and α-boswellic acids (αBAs) and their esters. The analysis of these triterpenes is performed by different analytical methods including High Performance Thin Layer Chromatography (HPTLC) [26], although the most used methods are based on HPLC coupled to both photodiode array detection [27] and mass spectrometry detection [28]. In accordance with the spectral properties of the boswellic acids, their analysis is performed at three different wavelengths, 210 nm for αBAs, βBAs as well as lupeolic acid, 250 nm for AKBA and KBA, and 280 nm for 9,11-dehydro-α- and -β-boswellic acids [27]. However, a precise identification and quantification of boswellic acids is usually obtained with liquid chromatography electrospray ionisation tandem mass spectrometry (LC-ESI-MS/MS) by using selected ion monitoring (SIM) detection [28].

In general, the total organic acids from *B. serrata* and *B. sacra* constitute approximately 65%–70% by weight of the total alcoholic extract. Of this fraction, approximately 25% is made of triterpenes. In market products, these percentages are often misinterpreted and it is not unusual to find claims of 70% boswellic acids content or 30% AKBA content, which is obviously misleading because the highest amounts so far reported of boswellic acids in *B. serrata* is about 140 mg/g (i.e., 14%) and in *B. sacra* is about 190 mg/g (i.e., 19%) [27]. Since both *Boswellia* extracts are used in several formulations, it is important to express unequivocally the “real content” of boswellic acids in both *B. serrata* and *B. sacra*. Therefore, the aim of this work is to provide a guideline for the accurate identification and quantification of boswellic acids in these two important *Boswellia* species by using HPLC-DAD-ESI-MS/MS.

## 2. Results and Discussion

### 2.1. Identification of Boswellic Acids

Gum resin of *B. sacra* and *B. serrata* were extracted using methanol in order to evaluate the total BAs content. The total yield for *B. sacra* methanolic extract was 598.88 g·kg^−1^ (±11.40 g·kg^−1^) gum resin dry weight whereas the yield for *B. serrata* was 549.78 g·kg^−1^ (±29.31 g·kg^−1^) gum resin dry weight. The total recovery of methanolic extracts from *B. sacra* was 99.17% (±1.93%), whereas the total recovery from *B. serrata* was 98.96% (±1.97%). Our findings indicate that the total content of the lipophilic extracts (excluding the polysaccharide moiety of both species) never exceeded 60%, in agreement with previous works [27,29,30]. By considering that the methanolic fraction contains several lipophilic compounds, including mono-, di- and triterpenes, it is evident that a claim of 70% BAs is not sustainable, being the BAs only a portion of the total methanolic extract.

**Figure 1:**
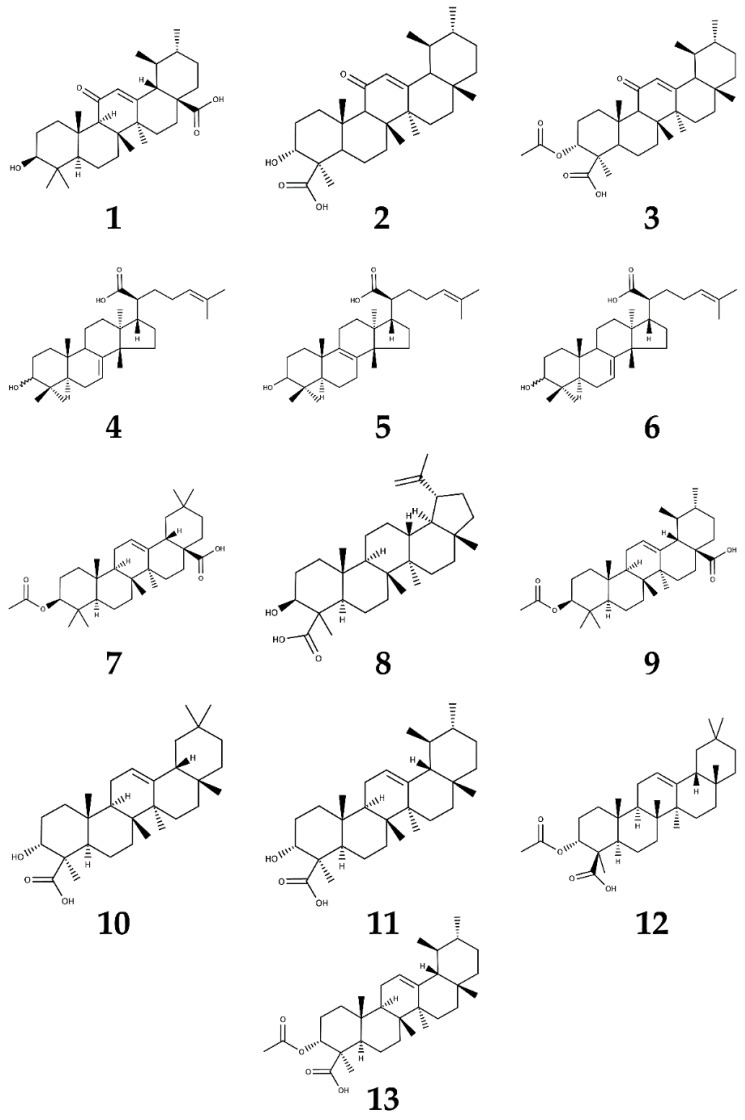
Chemical structure of the boswellic acids identified in *Boswellia serrata* and *Boswellia sacra*. Numbers correspond to compounds listed in Table 1.

In order to define the content and to identify the BAs present in the two Boswellia methanolic extracts, we performed HPLC-DAD-ESI-MS/MS analyses. Several BAs were present in both methanolic extracts. Table 1 reports the molecular mass and the fragmentation pattern of compounds identified in the methanolic extracts, whereas Figure 1 shows the chemical structure of the identified BAs. The identification of these compounds was achieved by both mass spectrometry and comparison with pure standards (see Supplementary Figure S1 for mass spectra of identified compounds and Supplementary Figure S2 for UV chromatograms). In both species, the main BAs were represented by AKBA, KBA, αBA, βBA, acetyl-αBA (A-αBA) and acetyl-βBA (A-βBA), in accordance with the literature data [2,31,32].

### 2.2. Quantification of Boswellic Acids

Table 2 shows for both species the quantitative determination of the main BAs. The total amount of the main BAs was statistically (*p* < 0.05) higher in *B. sacra* than in *B. serrata*. In *B. sacra,* the total amount of the main BAs in the methanolic extract was lower than 50% and this value was reduced to about 29% when the amount was considered in terms of the total gum resin dry weight (Table 2). In *B. serrata* the total content of the main BAs in the methanolic extract was lower than 30% and the value dropped to 16% when BAs were calculated in terms of the gum resin dry weight (Table 2). In both species, the major BAs were represented by αBA and βBA, in agreement with literature data [32]. A direct comparison between the two species shows that the contents of *B. sacra* AKBA (about 10 fold), αBA (1.5 fold) and βBA (1.6 fold) were statistically (*p* < 0.05) higher than in *B. serrata*.

In order to validate the quantitative analyses reported in Table 2, we calculated the linearity and precision of the identified BAs standard curves, the detection limit (LOD), the quantification limit (LOQ) and the Matrix Effect (ME). Table 3 shows the validation results for the main identified BAs. All compounds showed a high R^2^ value, which indicates a high linearity in the calibration curves. The lowest LOD and LOQ values were found for A-βBA, followed by equal values for KBA and AKBA. The highest LOD and LOQ values were found for A-αBA. In order to complete the validation process, we assessed the ME, to assure that precision, selectivity and sensitivity were not compromised during HPLC-ESI-MS/MS analyses. The absolute ME was calculated by comparing the slope of matrix-matched standard curve with the slope of the standard calibration curve, according to [33]. Table 3 reports, for each species, the ME accuracy (expressed as percent values) of the main identified BAs. In *B. sacra* extracts, KBA (CV = 14.06) and A-βBA (CV = 11.61) showed the highest percentage of accuracy, followed by AKBA (CV = 7.53) and A-αBA (CV = 1.90). βBA (CV = 8.72) showed the lowest ME accuracy percentages. In *B. serrata*, the highest percentages of accuracy were found for αBA (CV = 0.22), followed by KBA (CV = 14.13), A-αBA (CV = 2.66) and AKBA (CV = 7.74).

Finally, Table 4 shows the recovery of the identified BAs from *B. sacra* and *B. serrata* methanolic extracts. In both species, the total recovery was higher than 98%. In *B. sacra*, the highest recovery was found for AKBA and αBA, whereas in *B. serrata* the highest recovery was found for A-βBA and A-αBA (Table 4).

The quantitative determination of *B. sacra* and *B. serrata* BAs content and the validation of the quantitative chemical analysis show that any claim of BAs content in either *B. sacra* or *B. serrata* gum resins equal to or higher than 70% or 30% AKBA are simply unrealistic or based on a wrong quantitative determination. The same is true when the percentage of BAs is calculated in the methanolic extract.

## 3. Materials and Methods

### 3.1. Plant Material and Chemicals

*Boswellia serrata* Roxb. and *Boswellia sacra* Flueck gum resins were purchased from Bauer S.r.l. (Udine, Italy). The origin of *B. sacra* samples was from Ethiopia, whereas *B. serrata* samples originated from India. The gum resins were milled to coarse powder and used for all extractions. All chemicals were of analytical reagent-grade unless stated otherwise. Pure standards were purchased for the quantification by external calibration curves: 11-Keto-β-boswellic acid, α-Boswellic acid and β-Boswellic acid (ExtraSynthese, Lyon, France), 3-*O*-Acetyl-11-keto-β-Boswellic Acid (Merck, Darmstadt, Germany), 3-*O*-Acetyl-α-boswellic acid, 3-*O*-Acetyl-β-boswellic acid (Sigma-Aldrich, St. Louis, MO. USA).

### 3.2. Solvent Extraction of Boswellia serrata and Boswellia sacra oleo Gum Resins

One hundred grams of ground *B. serrata* and *B. sacra* oleo gum-resins were extracted with 1 L methanol (VWR International, Radnor, PA, USA) (extraction ratio 1:10 *w*/*v*). Samples were then placed on an orbital shaker for 5 days in the dark. Extracts were then filtered and the resin was rinsed with 400 mL of methanol. To evaluate the recovery of analyzed compounds, the exhaust gum resin was re-extracted with methanol as previously described. Samples were then concentrated by vacuum evaporation (Rotavapor, Büchi, Flawil, Switzerland). Concentrated extracts were then dried in a ventilated oven at 70 °C for 4 h. The powdered extracts were stored at room temperature in the dark until chemical analysis. Extractions were performed in triplicate.

### 3.3. Isolation and Quantification of Boswellic Acids by HPLC-DAD-ESI-MS/MS

Boswellic acids were identified and quantified by liquid chromatography (1200 HPLC, Agilent Technologies, Santa Clara, CA, USA) equipped with a reverse phase column, Luna C18 (3 µm, 150 mm × 3.0 mm, Phenomenex, Torrance, CA, USA). *B. serrata* and *B. sacra* powdered extracts were dissolved (30 mg·mL^−1^) in HPLC-grade methanol and properly diluted. The binary solvent system was: (A) MilliQ H_2_O (Millipore, Billerica, MA, USA):Methanol 50:50 containing 5 mM ammonium acetate (Sigma-Aldrich, USA); and (B) Methanol:1-Propanol (VWR International, Radnor, PA, USA) 80:20 containing 5 mM ammonium acetate. The chromatographic separation was carried out at constant flow rate (200 µL·min^−1^) with the following conditions: linear gradient from 30% to 50% of B in 2 min, then 80% of B in 35 min, then at 47 min B concentration was raised to 98%. The concentration of solvent B was maintained at 98% for 6 min. The initial mobile phase was re-established for 10 min before the next injection. The temperature of wellplate autosempler G1377A was set 4 °C while chromatography was carried out at constant temperature (30 °C) controlled by an Agilent 1100 HPLC G1316A Column Compartment.

Tandem mass spectrometry analyses were performed with a 6330 Series Ion Trap LC-MS System (Agilent Technologies, USA) equipped with an electrospray ionization source (ESI) operating in negative mode. The flow rate of nitrogen was set 325 °C and 5.0 L·min^−1^, while the Capillary Voltage was 1.5 kV. Helium was used as a collision gas.

Identification of *Boswellia* oleo gum resin compounds was performed by scan analyses with a 50–750 *m/z* scan range and by monitoring the absorption at 210, 250 and 280 nm. Quantitative analyses were performed by Multiple Reaction Monitoring (MRM) by monitoring the fragmentation of quasi-molecular ions for αBA and βBA and KBA (Table 1) and by Diode Array Detector (DAD) at 250 nm for AKBA and 210 nm for A-αBA and A-βBA. Quantification was performed by external calibration curves with pure standards dissolved in HPLC grade Methanol. Limit of Detections (LOD) and Limit of Quantifications (LOQ) for each compounds were determined as described in [34].

To evaluate the ME in the quantification of target compounds, *B. serrata* and *B. sacra* powdered extracts were dissolved (30 mg·mL^−1^) in HPLC-grade methanol and properly diluted. These sample solutions were used to prepare the calibration curves in the presence of other extracted gum resin compounds [33]. The slope of standard curves obtained with the solvent (methanol) and in the extracts were used to compare the ME percentage (%ME = Calibration Slope_(sample)_/CalibrationSlope_(standard)_ × 100). 100% ME percentage indicates no ME, a ME% < 100% indicates ionization suppression and a ME% > 100% indicates ionization enhancement.

## 4. Conclusions

*Boswellia sacra* and *Boswellia serrata* extracts are widely used in pharmaceutical and nutraceutical preparations. The bioactivity of these *Boswellia* extracts is based on the content of BAs. Clearly, the dose of bioavailable BAs is central to the issue of *Boswellia* efficacy. Claims of 70% BAs or even 30%–40% AKBA are currently found, but this work confirms that the BAs content never exceeds 50% of the methanolic extract, whereas lower percentages are obtained when BAs are expressed in terms of the gum resin weight. Moreover, the highest percentage of AKBA found in *B. sacra* was below 8%. Only analytical methods based on HPLC coupled to mass spectrometry allow the precise quantification and identification of BAs in *Boswellia* extracts, whereas other methods based only on HPLC or spectrophotometric methods do not sufficiently allow an accurate quantification of BAs. Therefore, we recommend LC-MS technology for BAs determination and quantification.

## Figures and Tables

**Table 1:** Molecular mass and fragmentation pattern of compounds identified in the methanolic extracts of *B. sacra* and *B. serrata*.

Compounds		RT (min)	[M − H]^−^(*m/z*)	Ions (*m/z*)	Occurrence in	
					*B. sacra*	*B. serrata*
**1**	11-Keto-Ursolic acid	24	469	451, 407, 391	x	x
**2**	11-Keto-β-boswellic acid	25.8	469	451, 407, 391	x	x
**3**	3-*O*-Acetyl-11-keto-β-Boswellic Acid	28.8	511	451, 361	x	x
**4**	3-α-Hydroxy-7,24-dien-tirucallic acid	34.5	455	453, 437, 373		x
**5**	3-α-Hydroxy-8,24-dien-tirucallic acid	36.5	455	453, 437, 373	x	x
**6**	3-β-Hydroxy-7,24-dien-tirucallic acid	39.4	455	453, 437, 373		x
**7**	3-*O*-Acetyl-oleanolic acid	41	497	479, 437	x	x
**8**	Lupeolic acid	41.6	455	437, 409		x
**9**	3-*O*-Acetyl-ursolic acid	43.3	497	479, 437	x	x
**10**	α-Boswellic acid	44.5	455	437, 409, 377	x	x
**11**	β-Boswellic acid	45	455	437, 409, 377	x	x
**12**	3-*O*-Acetyl-α-boswellic acid	52	497	459, 437	x	x
**13**	3-*O*-Acetyl-β-boswellic acid	52.9	497	459, 437, 395	x	x

**Table 2:** Quantitative determination of boswellic acids in *Boswellia sacra* and *Boswellia serrata* by High Performance Liquid Chromatography-Diode Array Detector coupled to ElectroSpray Ionization and tandem Mass Spectrometry (HPLC-DAD-ESI-MS/MS), by using calibration curves from pure standards. Data are expressed as g·kg^−1^ gum resin dry weight. (Standard deviation), in the same row, different letters indicate significant (*p* < 0.05) differences.

Compound	*Boswellia sacra*		*Boswellia serrata*	
	Content in the Methanolic Extract	Content in the Gum Resin	Content in the Methanolic Extract	Content in the Gum Resin
KBA	35.50 (1.26) ^a^	21.26 (0.76) ^b^	34.62 (2.57) ^a^	19.03 (1.41) ^c^
AKBA	70.81 (4.66) ^a^	42.41 (2.79) ^b^	7.35 (0.89) ^c^	4.04 (0.49) ^d^
αBA	184.34 (11.27) ^a^	110.40 (6.75) ^b^	126.00 (7.77) ^b^	69.27 (4.27) ^c^
βBA	186.19 (4.98) ^a^	111.50 (2.98) ^b^	113.21 (7.37) ^c^	62.24 (4.06) ^c^
A-αBA	5.40 (0.35) ^a^	3.23 (0.21) ^b^	2.92 (0.19) ^b^	1.60 (0.11) ^c^
A-βBA	9.95 (0.23) ^a^	5.96 (0.14) ^b^	11.33 (0.28) ^a^	6.23 (0.15) ^b^
Total	491.20 (14.75) ^a^	294.77 (8.83) ^b^	295.25 (23.00) ^b^	162.29 (12.64) ^c^

**Table 3:** Validation of boswellic acids (BAs) quantitative analyses of methanolic extracts from *B. sacra* and *B. serrata*. (Standard deviation).

Compound	Regression Equation	R^2^	LOD µg·µL^−1^	LOQ µg·µL^−1^	ME *B. sacra*	ME *B. serrata*
KBA	y = 13891880514x + 180215436.7	0.973	0.006	0.020	100.67 (14.17)	98.83 (13.96)
AKBA	y= 24732.09x − 143.76	0.997	0.006	0.021	99.91 (7.53)	91.53 (7.08)
αBA	y = 1349052089x + 8552264.58	0.989	0.008	0.028	88.52 (9.75)	139.80 (0.31)
βBA	y = 1549451236x + 1749204.16	0.995	0.013	0.042	73.40 (6.40)	77.25 (7.11)
A-αBA	y = 9915.15x − 127.16	0.995	0.017	0.058	93.27 (1.77)	92.91 (2.47)
A-βBA	y = 9179.53x − 101.22	0.995	0.003	0.010	100.23 (11.64)	90.70 (8.74)

R^2^, coefficient of determination; LOD, Detection limit; LOQ, Quantification limit; ME, matrix effect, expressed as percentage of accuracy.

**Table 4:** Percentage of recovery of identified BAs from *B. sacra* and *B. serrata* methanolic extracts. Values are expressed as percentage of recovery.

Compounds	*B. sacra*	*B. serrata*
KBA	96.53	99.06
AKBA	99.68	96.88
αBA	99.39	99.14
βBA	98.75	97.13
A-αBA	88.71	99.38
A-βBA	91.34	99.84
Total	98.68	98.41

## Supplementary Materials

The following are available online at http://www.mdpi.com/1420-3049/21/10/1329/s1, Figure S1: mass spectra of boswellic acids isolated in this study; Figure S2: UV chromatograms of boswellic acids isolated in this study.
