# Supplementary material for: Quantitative Determination of 3-O-Acetyl-11-Keto-β-Boswellic Acid (AKBA) and Other Boswellic Acids in Boswellia sacra Flueck (syn. B. carteri Birdw) and Boswellia serrata Roxb
Source: Molecules. 2016 Oct 6;21(10):1329. doi: 10.3390/molecules21101329 (PMC6273064; doi:10.3390/molecules21101329)
Supplement: Supplementary file 1 [file molecules-21-01329-s001.pdf]

Supplementary Materials: Quantitative Determination of 3-*O*-Acetyl-11-Keto-β-Boswellic Acid (AKBA) and Other Boswellic Acids in *Boswellia sacra* Flueck (syn. *B. carteri* Birdw) and *Boswellia serrata* Roxb

Giuseppe Mannino, Andrea Occhipinti and Massimo E. Maffei

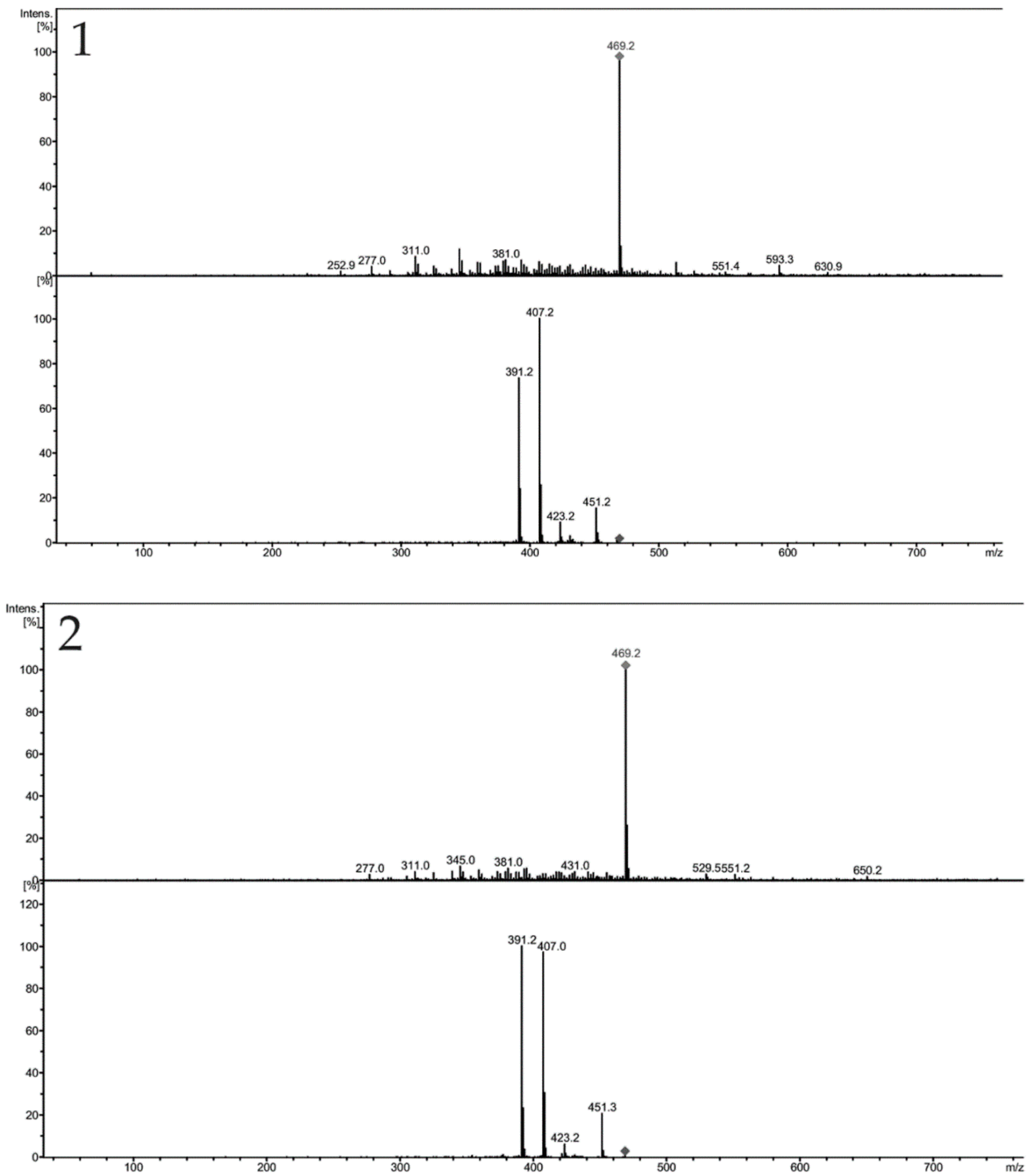

Figure S1. *Cont*.

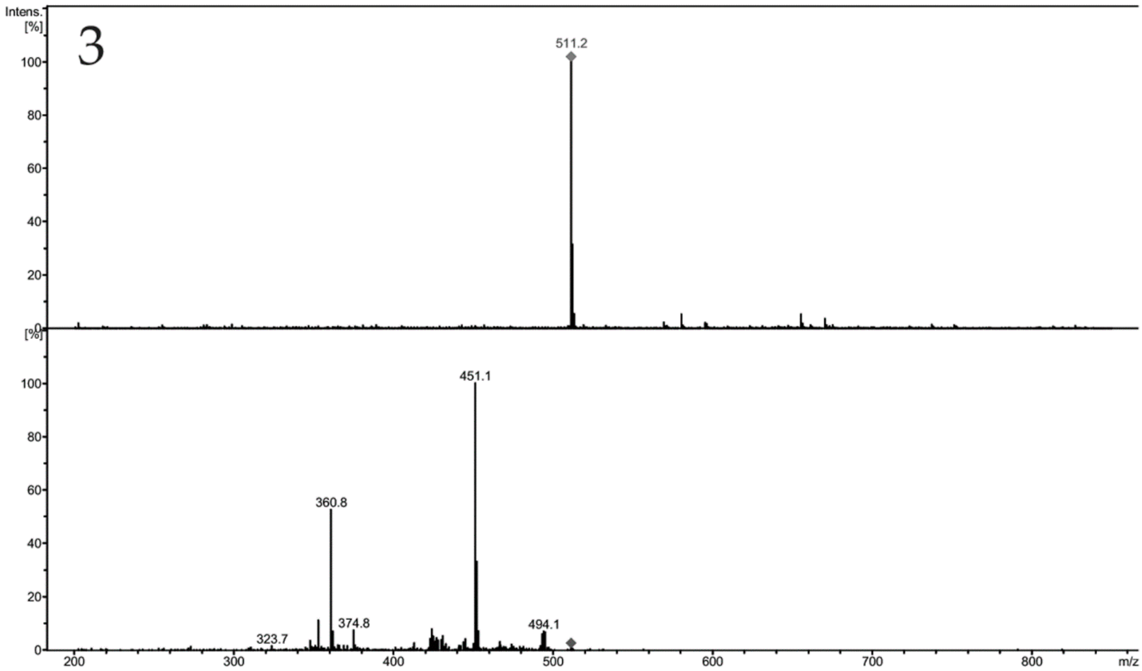

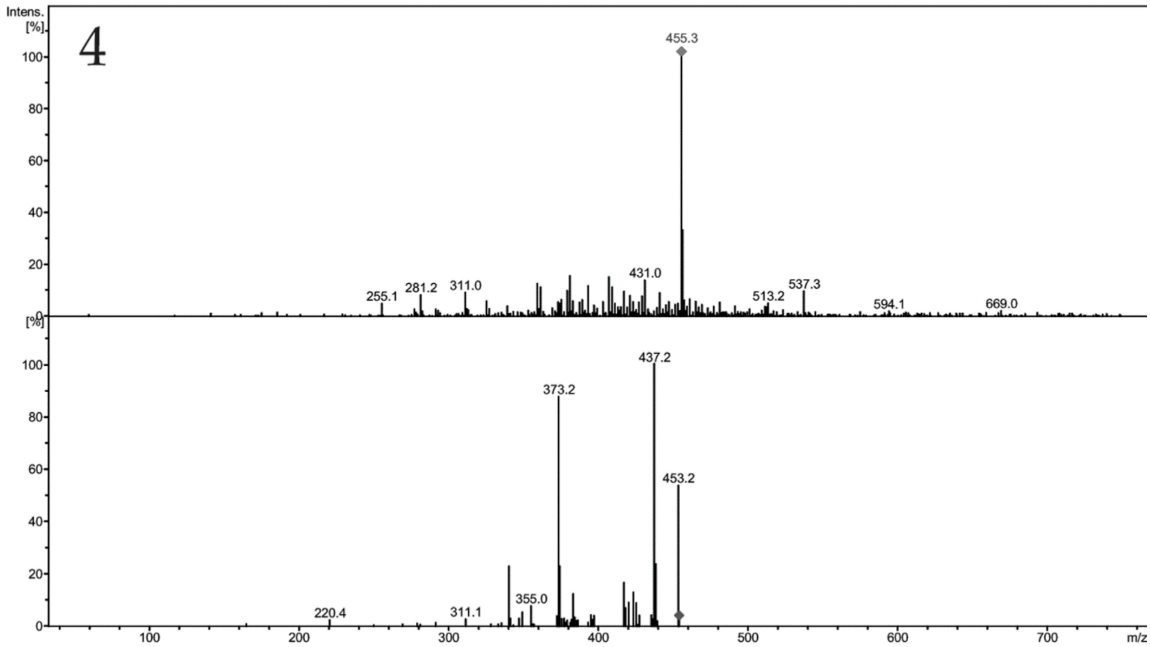

Figure S1. *Cont*.

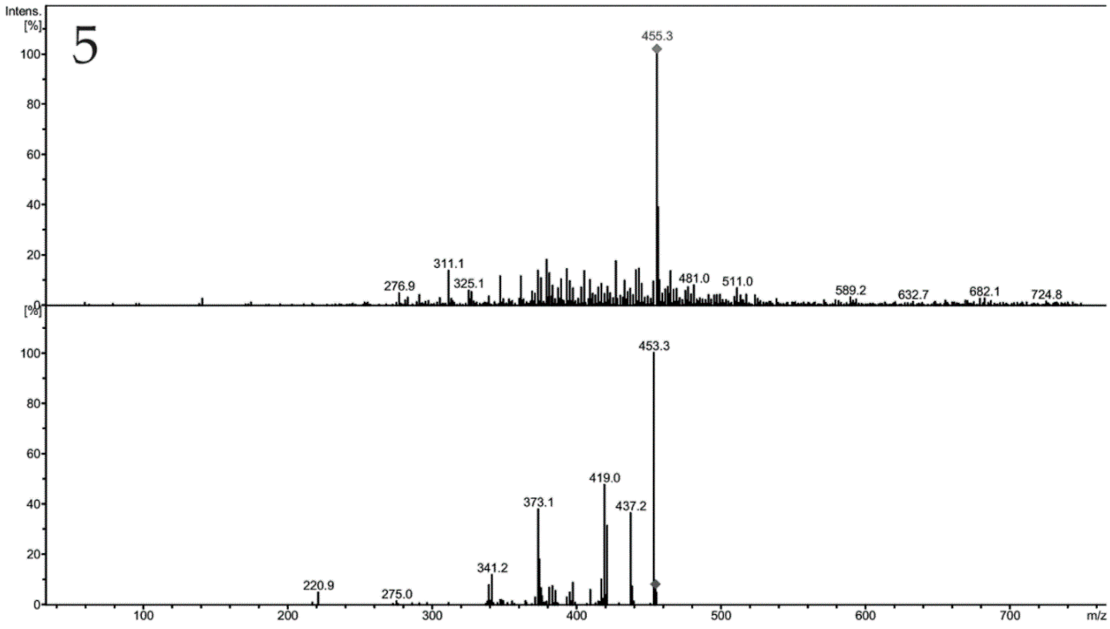

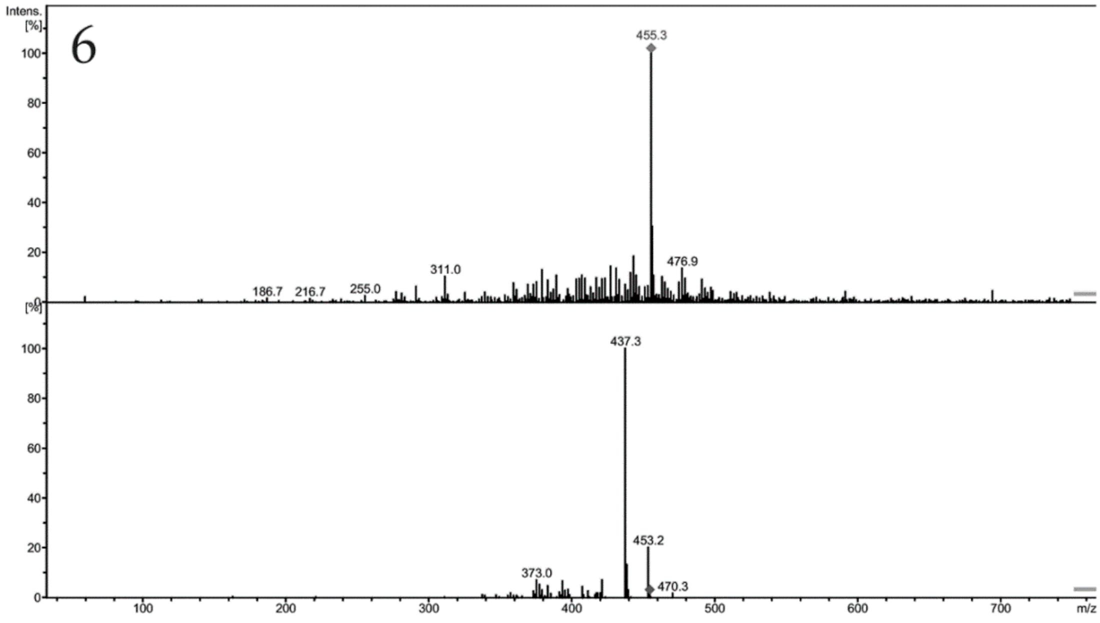

Figure S1. *Cont*.

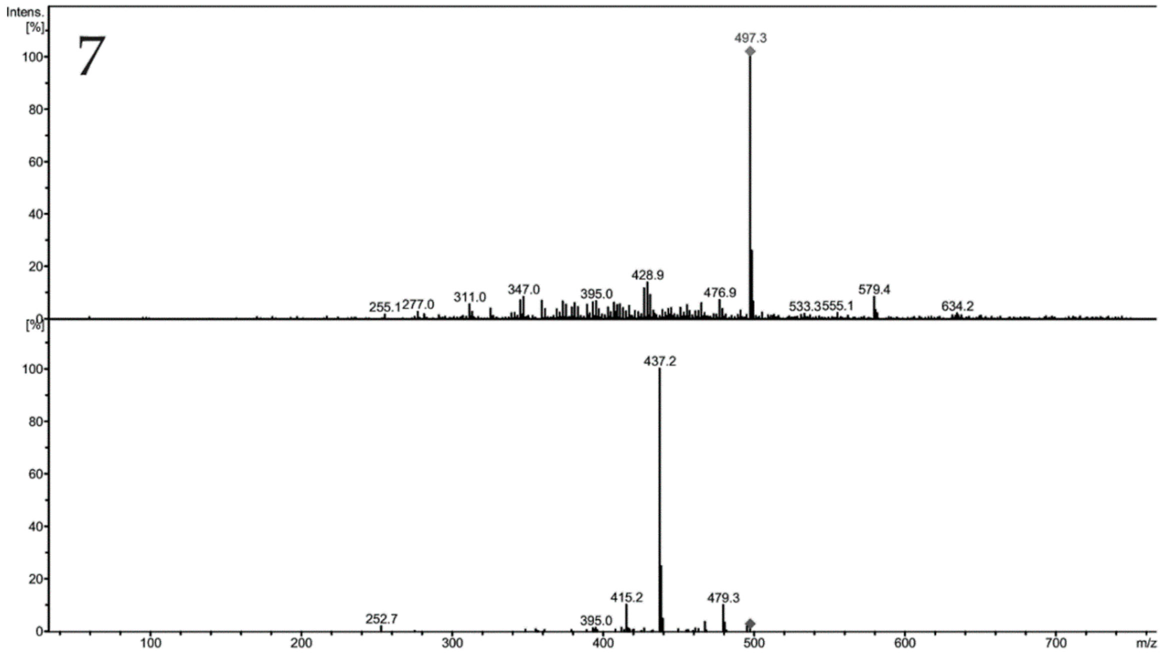

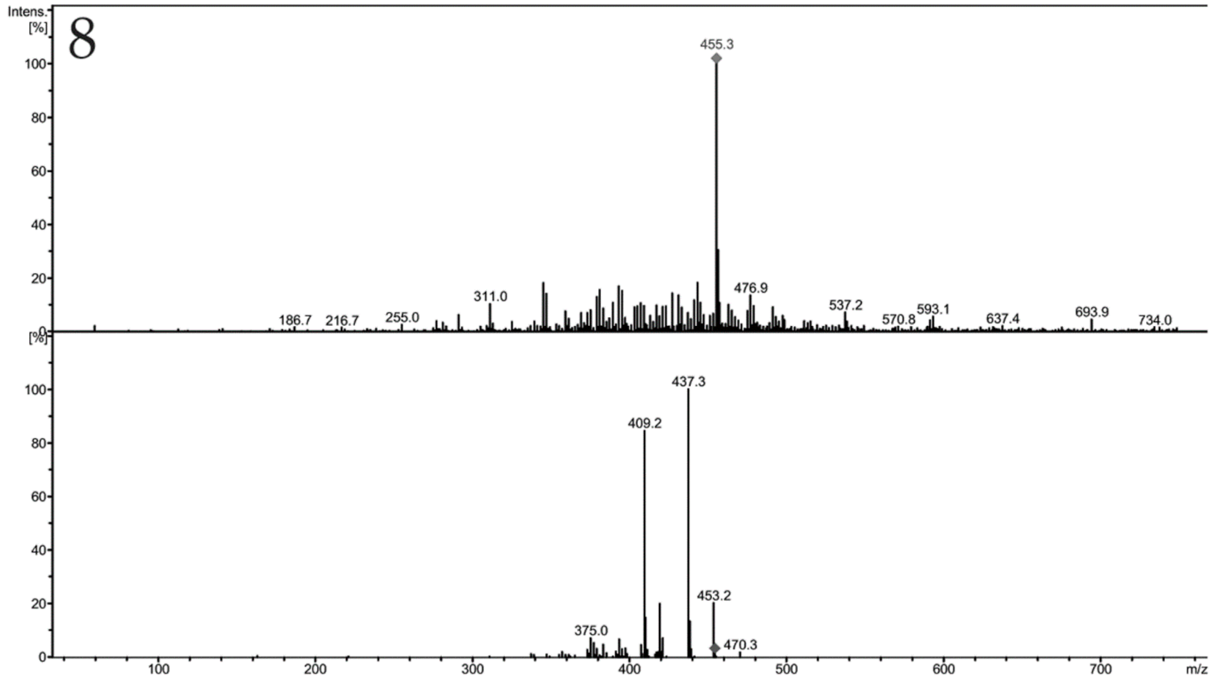

Figure S1. *Cont*.

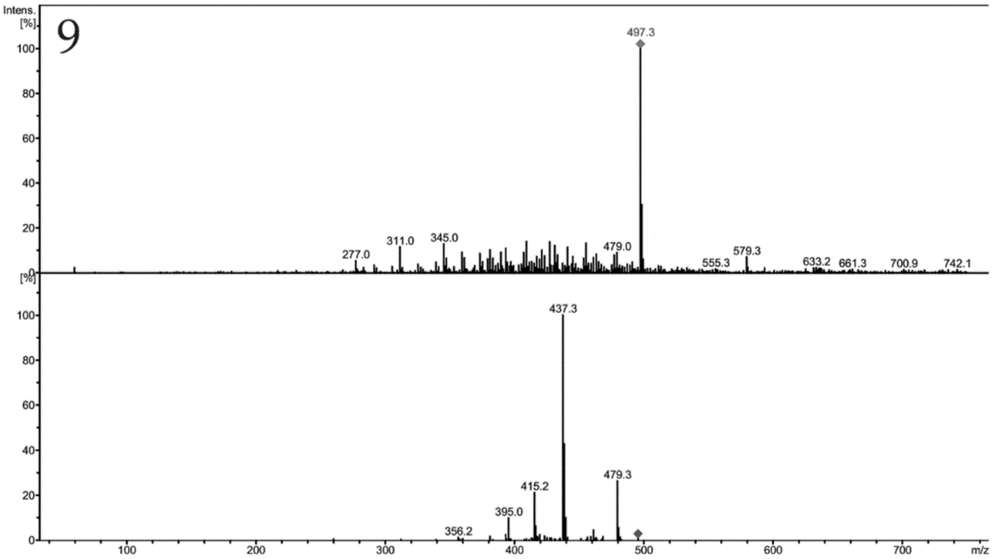

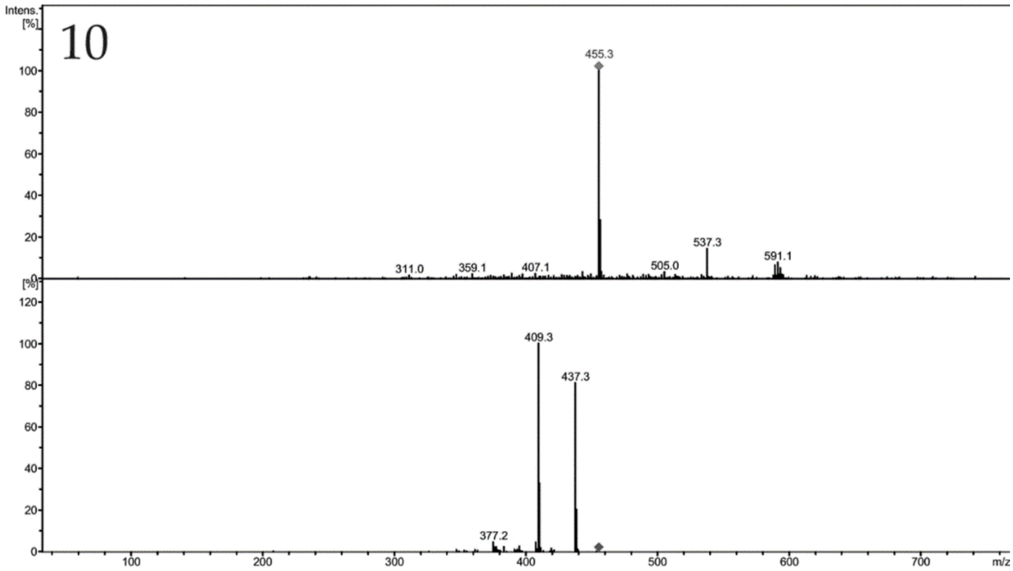

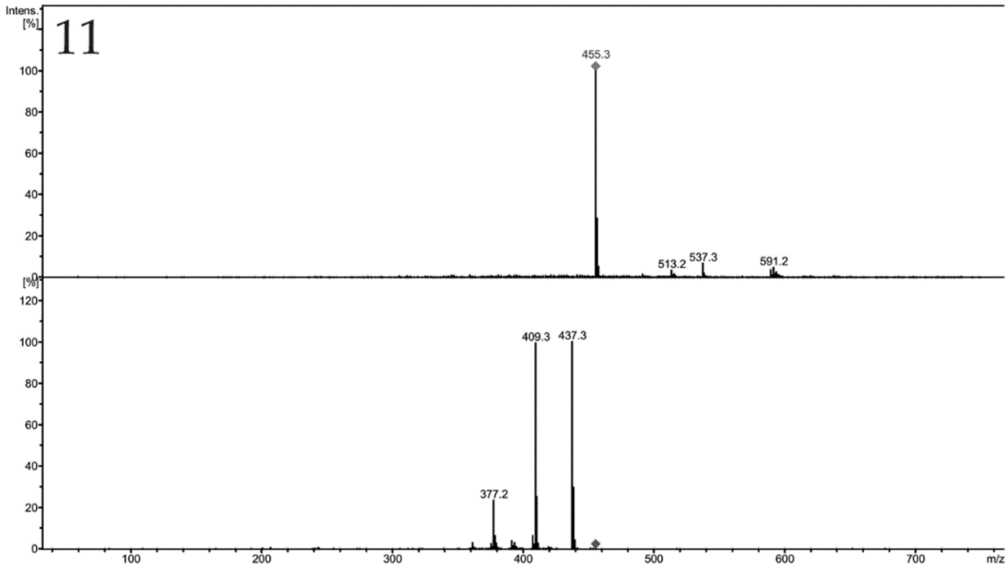

Figure S1. *Cont*.

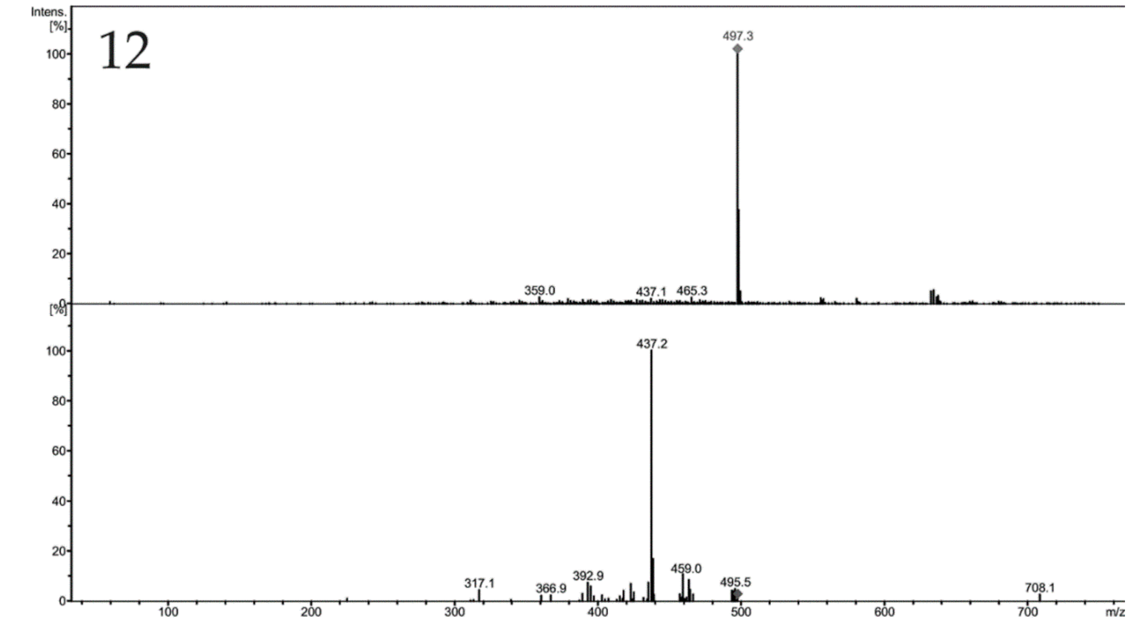

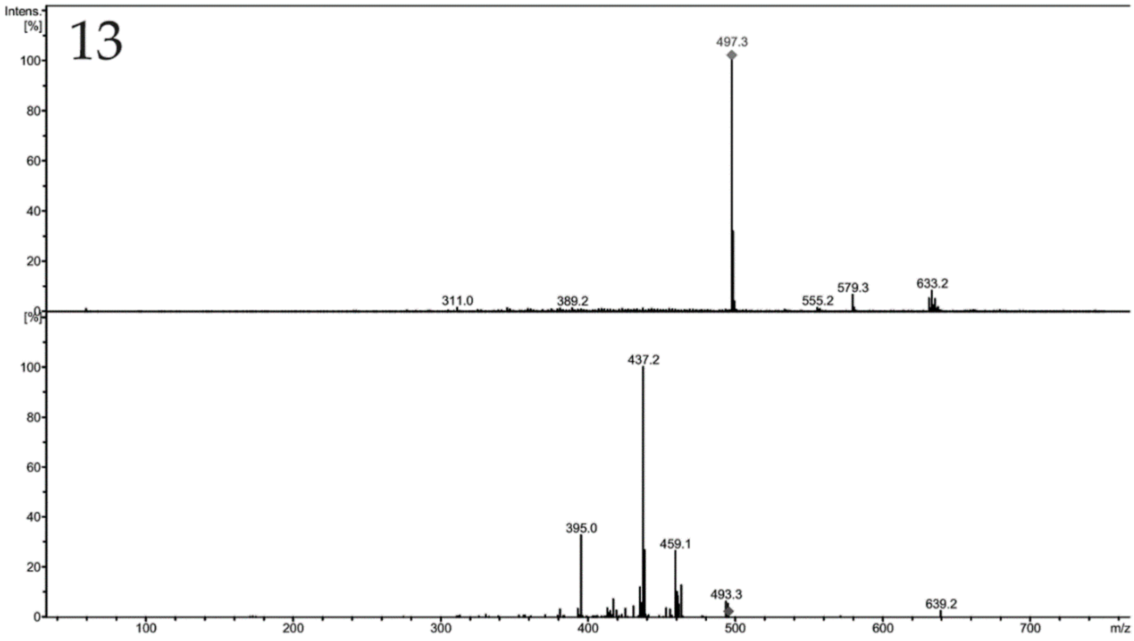

Figure S1. Mass spectra (MS_2) of identified boswellic acids. In each plate, upper left numbers correspond to molecules listed in Table 1.

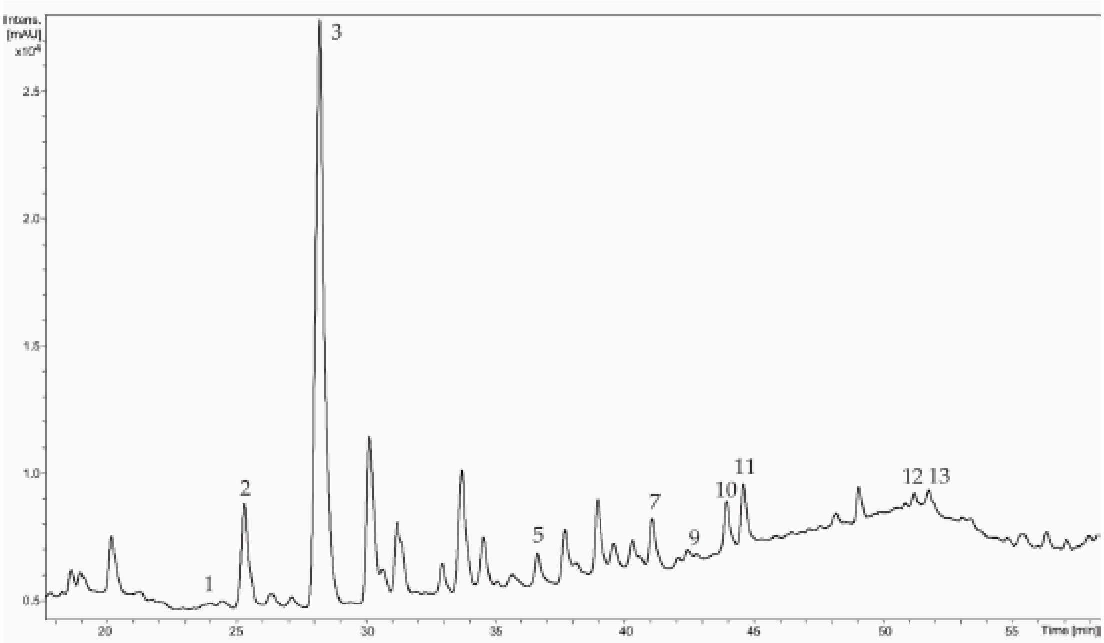

Chromatogram of *Boswellia sacra*. For chromatographic conditions, please refer to Materials and Methods. Peak numbers correspond to compounds listed in Table 1

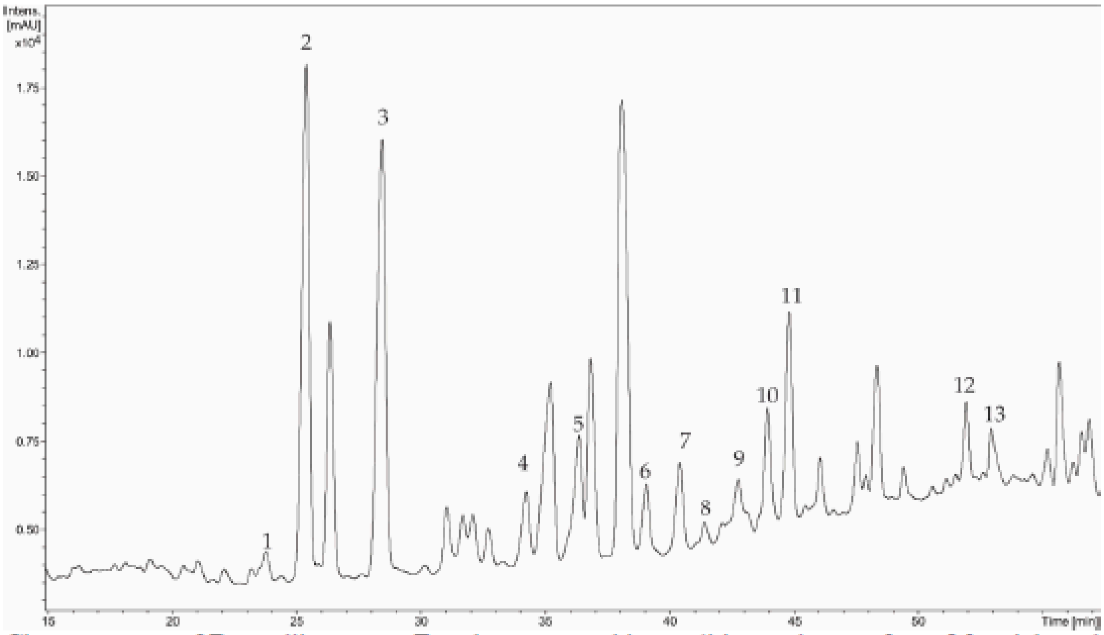

Chromatogram of Boswellia serrata. For chromatographic conditions, please refer to Materials and Methods. Peak numbers correspond to compounds listed in Table 1

Figure S2. UV-Chromatograms of Boswellia sacra and Boswellia serrate.
